# A Markov model assessing the cost-effectiveness of various anti-vascular endothelial growth factor drugs and panretinal photocoagulation for the treatment of proliferative diabetic retinopathy

**DOI:** 10.1038/s41433-025-03641-4

**Published:** 2025-02-05

**Authors:** Kirsty Luckham, Hannah Tebbs, Lindsay Claxton, Philip Burgess, Christiana Dinah, Noemi Lois, Syed Mohiuddin

**Affiliations:** 1 Science, Evidence and Analytics Directorate, National Institute for Health and Care Excellence, London, UK; 2 Department of Eye and Vision Science, University of Liverpool, Liverpool University Hospitals NHS Foundation Trust, Liverpool, UK; 3 Research and Innovation, London North West University Healthcare NHS Trust, London, UK; 4 School of Medicine, Dentistry and Biomedical Sciences, Queen’s University Belfast, Belfast, UK

**Keywords:** Health care economics, Retinal diseases

## Abstract

**Background:**

Proliferative diabetic retinopathy (PDR) may lead to vision loss and blindness. The cost-effectiveness of various anti-vascular endothelial growth factor (anti-VEGF) drugs and panretinal photocoagulation (PRP) was assessed to supplement the NICE guideline for treating PDR.

**Methods:**

A Markov model including eight levels of visual acuity (ranged between >85 and ≤25 letters) was developed to compare the cost-effectiveness of ranibizumab, aflibercept and bevacizumab with PRP (alone or in combination). Clinical inputs in the model were based on literature, while a published network meta-analysis (NMA) informed visual outcomes. Costs were estimated from a UK NHS perspective.

**Results:**

Assuming initial treatment effects from the NMA continued to be applied for the remainder of lifetime, the probabilistic analysis resulted in bevacizumab plus PRP producing the highest net monetary benefit (NMB [95% CI]) of £221,374 [£203,941–£238,388] at £20,000 per quality-adjusted life-year. However, assuming initial treatment effects stabilised over time resulted in PRP alone producing the highest NMB of £223,416 [£209,318–£236,866]. Results were associated with large uncertainty due to wide confidence intervals around vision-based treatment effects of anti-VEGFs versus PRP, particularly for bevacizumab as data were drawn from trials with small sample size and high risk of bias. Using confidential prices for aflibercept and ranibizumab did not change the overall findings.

**Conclusions:**

PRP is likely to be more cost-effective than anti-VEGFs for PDR. However, the results should be interpreted with caution given the scarcity of long-term visual outcomes with anti-VEGFs in this population. Further research on long-term visual outcomes may resolve these uncertainties.

## Introduction

Proliferative diabetic retinopathy (PDR) is a leading cause of blindness among working age adults in the UK [1] and worldwide [2], negatively affecting their quality-of-life (QoL) and ability to work [3]. The current standard of care for PDR is panretinal photocoagulation (PRP) with a proven track record of long-term stability of PDR regression [4, 5]. In the UK, PRP is delivered by doctors and has the potential of affecting the field of vision of people receiving this treatment. However, Protocol S [6] reported that, in PDR, a reduction in the field of vision occurs even when PRP is not applied. Intravitreal injections of anti-vascular endothelial growth factor (anti-VEGF) drugs have been approved by the National Institute for Health and Care Excellence (NICE) for treating diabetic macular oedema (DMO). However, anti-VEGFs have not yet been recommended by NICE for treating PDR, despite several randomised controlled trials (RCTs) showing some encouraging short-term results [6–8]. Anti-VEGFs can be administered by a range of healthcare professionals, but are generally expensive, require long courses of treatment, entail the risk of endophthalmitis, and there are concerns about their long-term effectiveness, cost-effectiveness and patient adherence in the context of treating PDR [9–11]. Furthermore, visual field loss may occur even when treated with an anti-VEGF [6].

The recent NICE guideline “Diabetic retinopathy: management and monitoring” [12] included a literature review to find economic evidence of anti-VEGFs and PRP for treating people with PDR. Prior to guideline development, only one study by Hutton et al. [13] met the inclusion criteria but this study was based on a short-time horizon and US population, so the cost-effectiveness conclusions were not seen as informative for the UK NHS due to the differences in healthcare systems. Since guideline development began, Walton et al. [10] assessed the cost-effectiveness of anti-VEGFs against PRP for treating PDR, indicating that the use of anti-VEGFs are unlikely to be cost-effective in this population. However, Walton et al. [10] did not include anti-VEGF as a combination option with PRP despite evidence of clinical efficacy [8], and considered ranibizumab, aflibercept and bevacizumab as a therapeutic class and compared this with PRP alone. Other economic analyses have assessed PRP and anti-VEGFs, but these were cost evaluation only [14] or in a non-PDR population [15].

The NICE guideline committee on diabetic retinopathy were tasked with determining the most clinically effective and cost-effective interventions for the management of PDR in a UK NHS setting. The committee, comprising of, among others, consultant ophthalmologists, diabetologists and patient members, directed the NICE guideline development team to build a de novo cost-effectiveness model. Markov models are useful in NICE decision-making process, where transparency is paramount and accessibility is required to allow a range of stakeholders to critically appraise the model. Therefore, this study used a simple and flexible Markov model structure as opposed to a more complex discrete event simulation (DES), to compare the cost-effectiveness of various anti-VEGFs with PRP (alone or in combination with an anti-VEGF) within the same analysis for treating people with PDR without DMO. Cross-model validation is a crucial process to ensure cost-effectiveness models are robust and valid for decision-making [16]; as such, the benefits and challenges of using simple and complex models to address a similar question were also discussed in this study.

## Methods

### Model overview and structure

A cohort Markov model with a 3-monthly cycle length (and a half-cycle correction) was developed in Microsoft® Excel® to compare the lifetime costs and quality-adjusted life-years (QALYs) of ranibizumab (Lucentis) 500 µg, aflibercept (Eylea) 2 mg and bevacizumab (off-label) 1.25 mg against PRP (either alone or in combination with an anti-VEGF) for treating people with PDR without DMO. Ranibizumab biosimilar (Ongavia) 500 µg was considered in a separate analysis assuming the same efficacy, safety and resource use as ranibizumab (Lucentis). The analysis was conducted from the perspective of UK NHS and personal social services, and all costs and QALYs were discounted at 3.5% per year [17].

**Fig. 1:**
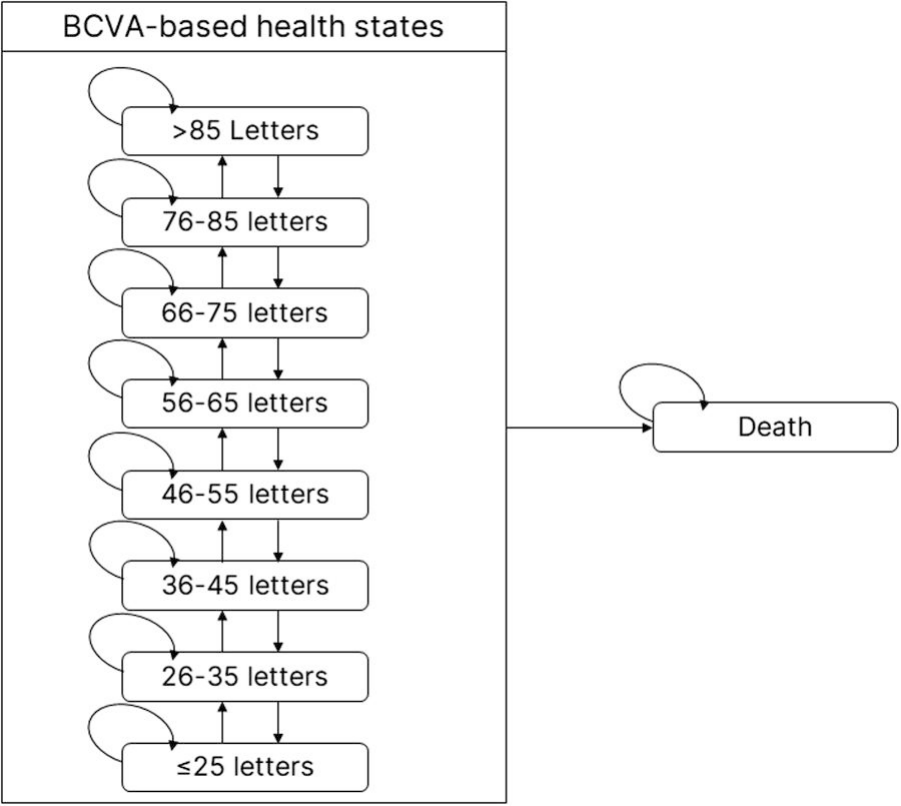
Schematic diagram of the Markov model showing possible health state transitions. Markov Model structure diagram of nine health states involving eight levels of best corrected visual acuity (BCVA) and a death state.

Shown in Fig. 1, the Markov model structure included nine health states involving eight levels of best corrected visual acuity (BCVA) and a death state. Informed by clinical input, the model allowed people to move by up to one BCVA state in each 3-monthly cycle, i.e. an increase or decrease in BCVA by between 5 and 15 letters (10-letter range) to account for the average eye rather than using the midpoint. The rationale for using a model based on BCVA (even though it may not fully capture all outcomes associated with disease progression) was to allow for the main outcome data reported from RCTs to be incorporated into the model and the results to be validated against previously published cost-effectiveness analyses.

### Baseline data

The model cohort started at the age of 56 years; 57.6% were male [18]. The model assumed a starting distribution of people across BCVA-based health states at baseline (Table 1). Aligned with previously published BCVA-based models [19–22], the model structure was based on one eye only. However, as was done in NICE TA799 [23] and NICE TA820 [24], the costs associated with treatment of the second eye were also included based on the assumption that 22% of people had treatment in both eyes at baseline [22]. For those with treatment in one eye, 67.2% had treatment in their worst seeing eye (WSE) and 32.8% had treatment in their best seeing eye (BSE) [20]. The probability of developing disease in the second eye was 5.4% per 3-monthly cycle [21], and it was assumed that the same treatment would be used in both eyes. The parameters used in the model are shown in Table 1.

**Table 1:** Various parameters used in the model.

Parameter	Point estimate	Probabilistic analysis		Sources
		Distribution	Parameters	
Baseline starting distribution of BCVA in each health state for treated eye				
BCVA: >85	0%	Dirichlet	N/A	Régnier et al. [22]
BCVA: 76–85	11%			
BCVA: 66–75	39%			
BCVA: 56–65	27%			
BCVA: 46–55	15%			
BCVA: 36–45	8%			
BCVA: 26–35	0%			
BCVA: ≤25	0%			
Fellow eye involvement				
Probability of DMO in the fellow eye	5.4%	Beta	*α* = 7.900, *β* = 138.397	Pochopien et al. [21]
Patients treated in both eyes	22.0%	Beta	*α* = 75.900, *β* = 269.100	Régnier et al. [22]
Patients treated in WSE	67.2%	Dirichlet	N/A	Mitchell et al. [20]
Patients treated in BSE	32.8%			
Natural history				
Mean change in ETDRS (52 weeks)	−1.300	Normal	*μ* = −1.300, *σ* = 0.364	Maturi et al. [18]
Probability of gaining one health state (SHAM)	2.6%	N/A	N/A	Calculated
Probability of losing one health state (SHAM)	6.1%			
Mortality hazard ratio (HR) for diabetes				
Mortality HR diabetes	1.950	Lognormal	*μ *= 0.668, *σ* = 0.090	Preis et al. [31]
Treatment effect at 1 year (mean difference, LogMAR)^a^				
Aflibercept (vs PRP)	−0.088	Normal	*μ* = −0.088, *σ* = 0.070	Simmonds et al. [25]
Ranibizumab (vs PRP)	−0.123		*μ* = −0.123, *σ* = 0.058	
Ranibizumab plus PRP (vs PRP)	−0.080		*μ* = −0.080, *σ* = 0.042	
Bevacizumab (vs PRP)	−0.193		*μ* = −0.193, *σ* = 0.499	
Bevacizumab plus PRP (vs PRP)	−0.172		*μ* = −0.172, *σ* = 0.055	

*BCVA* best corrected visual acuity,* BSE* best seeing eye,* DMO* diabetic macular oedema,* ETDRS* early treatment diabetic retinopathy study,* N/A* not applicable,* LogMAR* logarithm of the minimum angle of resolution,* PRP* panretinal photocoagulation,* WSE* worst seeing eye.

^a^*PDR* clinical trials did not have a no treatment or sham arm, so treatment effects for all interventions were compared with *PRP*.

### Treatment effectiveness and transition probabilities

Treatment effects were derived from a published network meta-analysis (NMA) [25] that reported the 1-year mean change in BCVA, relative to PRP, including fourteen RCTs (three of aflibercept, five of bevacizumab and six of ranibizumab). The RCTs that fed into the NMA are summarised in the paper [25]. The probability of transitioning between the different health states were estimated assuming that the changes in BCVA are normally distributed [26]. For PRP, the mean BCVA change from baseline to 1 year was used as the weighted average of that reported in the CLARITY [8] and PRIDE [27] trials (Table S1; Supplementary material); this was then converted to LogMAR to calculate the 3-monthly probabilities of 4.79% of gaining one health state and 8.48% of losing one health state (Table S2; Supplementary material).

The model explored two scenarios: (i) 1-year BCVA-based treatment effects from the NMA were applied for the first year and assumed to continue to be applied for the remainder of the lifetime for all interventions, and (ii) 1-year BCVA-based treatment effects from the NMA were applied for the first year for all interventions, and then stabilised beyond the first year for PRP and beyond the second year for anti-VEGFs with a linear decline between the first and second years based on the 5-year outcomes reported in Protocol S [6].

### Treatment discontinuation

The model assumed that 100% of people would remain on treatment for the first year (clinical consensus), 87% would remain on treatment from 1 to 3 years [7], 75% would remain on treatment from 3 to 5 years [28] and 50% would remain on treatment from year 5 onwards [23]. Treatment discontinuation was assumed to be the same across all interventions.

### Natural history

Data from the sham arm of Protocol W [18] in a non-proliferative population was used to inform natural history after the efficacy duration of treatments was assumed to have ended; natural history data was only required to conduct some deterministic sensitivity analyses. A mean (SD) 1-year change in BCVA of −1.30 (4.90) ETDRS letters [18] was used to calculate the 3-monthly probabilities of 2.57% of gaining one health state and 6.10% of losing one health state for the long-term natural history of PDR (Table 1). Although a non-proliferative population may not be fully representative of proliferative disease, clinical consensus was that the use of Protocol W [18] would be an acceptable proxy.

### Adverse events (AEs)

The reporting of treatment related AEs across RCTs varies considerably. It was still considered important to include AEs where possible. In the absence of data, the frequency of AEs for ranibizumab plus PRP, bevacizumab, or bevacizumab plus PRP was assumed to be equivalent to ranibizumab, as agreed by the clinician members of the NICE diabetic retinopathy guideline committee. The proportion of each AE by treatment is shown in Table S3 (Supplementary material).

### Mortality

Mortality was modelled using age- and gender-specific National life tables for England and Wales (2018–2020) [29]. The main mortality risk associated with poor vision was expected to be captured in the diabetes population rather than in diabetic retinopathy population. As such, similarly to NICE TA346 [30], a hazard ratio of 1.95 [31] was applied to account for the increased mortality risk associated with diabetes relative to the general population.

### Resource use and costs

The costs associated with treatment (list prices), administration, monitoring, and low vision (BCVA ≤ 35 letters) are shown in Table 2. Cost year 2019-20 was used to represent usual care in the NHS and to avoid any cost outliers due to the COVID-19 outbreak.

**Table 2:** Cost and utility parameters used in the model.

Cost/Utility	Point estimate	Probabilistic analysis^a^		Sources/Notes
		Distribution	Parameters	
Treatment cost (list price)				
Aflibercept (Eylea) 4.0 mg	£816.00	Gamma	*μ* = 96.036, *σ* = 8.497	BNF 28/03/2023 bnf.nice.org.uk
Ranibizumab (Lucentis) 2.3 mg	£551.00		*μ* = 96.036, *σ* = 5.737	
Ranibizumab biosimilar (Ongavia) 2.3 mg	£523.45^b^		*μ* = 96.036, *σ* = 5.451	
Bevacizumab 1.25 mg	£50.00^c^		*μ* = 96.036, *σ* = 0.521	NICE TA824 [35]
Panretinal photocoagulation (PRP)	£126.77		*μ* = 96.036, *σ* = 1.320	NICE TA346 [30], NHS reference cost 2019–20, HRGs code BZ87A minor vitreous retinal procedures.
Administration cost for anti-VEGFs				
Optical coherence tomography (applied to 100% of visits)	£101.804	Gamma	*μ* = 96.036, *σ* = 1.060	NHS reference costs 2019–20. Consultant led non-admitted face-to-face attendance, follow-up. Code 130 (ophthalmology). Assumption used in NICE TA294 [45].
Administration visit – outpatient (applied to 95% of visits)	£129.616		*μ* = 96.036, *σ* = 1.350	NHS reference costs 2019–20. Outpatient procedure. BZ87A minor vitreous retinal procedures. Assumption used in NICE TA294 [45].
Administration visit - day case (applied to 5% of visits)	£660.838		*μ* = 96.036, *σ* = 6.881	NHS reference costs 2019–20. Day case procedure. BZ87A minor vitreous retinal procedures. Assumption used in NICE TA294 [45].
Anti-VEGF administration per visit	£257.981	N/A	N/A	Calculation based on above inputs.
Monitoring cost				
Monitoring visit during treatment	£101.804	Gamma	*μ *= 96.036, *σ* = 1.060	NHS reference costs 2019–20. Consultant led non-admitted face-to-face attendance, follow-up. Code 130 (ophthalmology). Assumption used in NICE TA294 [45].
Monitoring visit post treatment	£38.344		*μ* = 96.036, *σ* = 0.399	£32 (2012–13) from Scanlon et al. [46] was inflated to 2019–20 prices.
Low vision cost per 3-monthly cycle				
Healthcare costs for low vision	£421.609	Gamma	*μ* = 25.003, *σ* = 16.862	Régnier et al. [22] Yearly total cost of visual impairment (BCVA ≤ 35) of £17,326 minus the cost of residential care (£15,327), community care (£600) and low vision rehabilitation (£47), to be aligned with NHS perspective. The costs (2010-11) were inflated to 2019-20 prices and then changed to 3-monthly cycle length.
Utility for best seeing eye (treated eye)				
BCVA: >85	0.839	Beta	*α* = 42.697, *β* = 8.193	Brown et al. [32]
BCVA: 76–85	0.839		*α* = 42.697, *β* = 8.193	
BCVA: 66–75	0.783		*α* = 141.181, *β* = 39.127	
BCVA: 56–65	0.783		*α* = 141.181, *β* = 39.127	
BCVA: 46–55	0.732		*α* = 44.858, *β* = 16.423	
BCVA: 36–45	0.681		*α* = 46.286, *β* = 21.682	
BCVA: 26–35	0.630		*α* = 45.992, *β* = 27.011	
BCVA: ≤25	0.579		*α* = 3.604, *β* = 2.621	
Utility for worst seeing eye (treated eye)				
BCVA: >85	0.839	Beta	*α* = 42.697, *β* = 8.193	The utility value for BCVA > 85 state for the worst seeing eye was set equal to the value for BCVA > 85 for the best seeing eye from Brown et al. [32]. Similar to the approach used by Régnier et al. [22], a utility decrement of 0.1 was assumed between the best (BCVA > 85) and worst (BCVA ≤ 25) health states, and a linear decline was assumed for calculating the utility values for the other states.
BCVA: 76–85	0.839		*α *= 42.697, *β* = 8.193	
BCVA: 66–75	0.822		*α* = 45.330, *β* = 9.794	
BCVA: 56–65	0.806		*α* = 47.651, *β* = 11.494	
BCVA: 46–55	0.789		*α* = 49.669, *β* = 13.283	
BCVA: 36–45	0.772		*α* = 51.396, *β* = 15.150	
BCVA: 26–35	0.756		*α* = 52.841, *β* = 17.085	
BCVA: ≤25	0.739		*α* = 54.016, *β* = 19.077	

*BCVA* best corrected visual acuity, *BNF* British National Formulary, *HRGs* healthcare resource groups, N/A not applicable, *NICE* National Institute for Health and Care excellence, *TA* technology appraisal.

^a^Varied by ±20% where relevant data were not available.

^b^This was only used in a scenario analysis assuming the same efficacy, safety and resource use as ranibizumab.

^c^This cost of £50 per 1.25 mg dose was used since this is around the price clinics would pay and is also aligned with previous TA.

Aflibercept (Eylea), ranibizumab (Lucentis) and ranibizumab biosimilar (Ongavia) have confidential patient access schemes which were available to NICE; these prices and analyses using them cannot be published due to confidentiality. A weighted cost of £257.98 was applied for administering an anti-VEGF (Table 2). It was assumed that the administration of PRP was captured within the cost of PRP itself. It was also assumed that treatment for both eyes would be administered in the same visit. Treatment monitoring costs included the cost of an optical coherence tomography scan in addition to the cost of a monitoring visit post treatment (Table 2). The number of monitoring visits for each treatment are shown in Table S4 (Supplementary material). The number of anti-VEGF injections per year are shown in Table S5 (Supplementary material); the same number of injections were assumed for all anti-VEGFs. The number of PRP treatments are shown in Table S6 (Supplementary material). All combination treatment options were assumed to have the same number of PRP treatments. The total cost of AEs for each treatment was calculated by multiplying the proportion of people experiencing an AE (Table S3; Supplementary material) with the cost of that AE (Table S4; Supplementary material).

Some people may switch onto another treatment after discontinuing the previous treatment due to a lack of response, but there is a large variability in reporting on subsequent treatments in the literature, and no data for the duration of subsequent treatments. The model allowed some people to receive a subsequent treatment based on the literature and clinical consensus (Table S9; Supplementary material), but this was applied to costs for two years only, to avoid overweighting the cost of first line treatment by the subsequent treatment for which the evidence was limited.

### Health state utility values

QoL data were derived from a review of utility literature and summarised in Table 2. Although a number of studies estimated utility values based on BCVA, the utility values for BSE for the diabetic retinopathy population from Brown et al. [32] were deemed to be most applicable, also used in NICE NG82 [33], NICE TA301 [34] and NICE TA824 [35], among others. Similarly to Haig et al. [19], the utility values for BSE were used for people who had treatment in both eyes since BSE is reported to be the major driver of overall QoL and their functioning [20, 36]. In addition to the health state utility values, utility losses for AEs (Table S8; Supplementary material) were included in the model.

### Deterministic and probabilistic sensitivity analyses

The following deterministic sensitivity analyses were explored for both BCVA-based treatment effect scenarios:

- Treatment occurs in a separate visit to monitoring.
- 25% of patients continue to receive treatment after 5 years.
- 75% of patients continue to receive treatment after 5 years.
- Natural history of PDR starts from 20 years.
- Natural history of PDR starts from 10 years.
- Natural history of PDR starts from 5 years.

The probabilistic sensitivity analysis was run to quantify uncertainty in the true values of input parameters. Probability distributions were specified for all input parameters and the type of distribution used was based on the properties of data of that type. Where possible, each distribution was parameterised using dispersion data from the source from which the value was obtained. Where no such data were available, plausible ranges were applied based on expert opinion.

## Results

The probabilistic lifetime cost-effectiveness results using list prices are shown in Table 3 for the two treatment effect scenarios. In the first scenario, 1-year treatment effects from the NMA continued to be applied for the remainder of the lifetime, which resulted in bevacizumab plus PRP having the highest net monetary benefit (NMB) (£221,374), bevacizumab alone having the second highest NMB (£216,410) and PRP alone having the third highest NMB (£212,190) at the £20,000 per QALY gained threshold. Using the incremental cost-effectiveness ratio (ICER) statistic, none of the anti-VEGFs alone were found to be cost-effective in the fully incremental analysis, while bevacizumab plus PRP had an ICER of £8,947 per QALY.

**Table 3:** Probabilistic cost-effectiveness results using list prices^a^.

Strategy	Total cost per person	Total QALYs^b^ per person	ICER^c^	NMB^d^ [95% CI] at £20,000 per QALY
Initial treatment effects were allowed to continue for the remainder of lifetime				
PRP	£8493	11.034	–	£212,190 [£196,602– £225,597]
Bevacizumab	£12,615	11.451	Extendedly dominated	£216,410 [£183,744– £239,858]
Bevacizumab plus PRP	£15,926	11.865	£8947	£221,374 [£203,941– £238,388]
Ranibizumab (Lucentis)	£26,435	11.673	Dominated	£207,018 [£188,241– £224,329]
Ranibizumab (Lucentis) plus PRP	£30,870	11.515	Dominated	£199,430 [£180,774– £215,929]
Ranibizumab biosimilar (Ongavia)^e^	£25,528	11.704	Dominated	£208,553 [£190,578– £224,770]
Ranibizumab biosimilar (Ongavia)^e^ plus PRP	£29,976	11.527	Dominated	£200,573 [£183,415– £218,262]
Aflibercept (Eylea)	£32,114	11.565	Dominated	£199,180 [£176,962– £218,849]
Initial treatment effects stabilised over time based on Protocol S				
PRP	£6517	11.497	–	£223,416 [£209,318– £236,866]
Bevacizumab	£11,677	11.549	Extendedly dominated	£219,303 [£203,877– £234,198]
Bevacizumab plus PRP	£15,714	11.651	£59,517	£217,309 [£202,512– £230,714]
Ranibizumab (Lucentis)	£26,028	11.603	Dominated	£206,041 [£191,991– £219,855]
Ranibizumab (Lucentis) plus PRP	£30,206	11.555	Dominated	£200,886 [£186,711– £215,028]
Ranibizumab biosimilar (Ongavia)^e^	£25,212	11.608	Dominated	£206,942 [£194,118– £220,788]
Ranibizumab biosimilar (Ongavia)^e^ plus PRP	£29,454	11.562	Dominated	£201,795 [£188,287– £215,375]
Aflibercept (Eylea)	£31,457	11.601	Dominated	£200,566 [£185,554– £215,098]

*ICER* incremental cost-effectiveness ratio, *NMB* net monetary benefit, *PRP* panretinal photocoagulation, *QALYs* quality-adjusted life years.

^a^Using the confidential patient access scheme prices for aflibercept, ranibizumab (Lucentis) and ranibizumab biosimilar (Ongavia) did not change the overall conclusions.

^b^QALYs were accrued by weighting the time spent in a health state with the corresponding utility value for that state and adjusting for the utility losses due to adverse events associated with treatment.

^c^Fully incremental analysis.

^d^NMB was calculated as [(QALYs × £20,000) − Cost], the strategy with the maximum NMB is deemed to be the most cost-effective.

^e^This was only used in a scenario analysis assuming the same efficacy, safety and resource use as ranibizumab (Lucentis).

In the second scenario, 1-year treatment effects from the NMA were applied in the first year for all interventions and then were allowed to stabilise over time based on Protocol S. This second scenario resulted in PRP producing the highest NMB (£223,416) at the £20,000 per QALY gained threshold. Bevacizumab plus PRP had an ICER of £59,517 per QALY, which is higher than NICE’s cost-effectiveness threshold, due to fewer QALY gains compared with the first scenario as expected. Using confidential prices for aflibercept and ranibizumab did not change the overall conclusions for both treatment effect scenarios.

Table 4 shows a summary of the probabilistic cost-effectiveness results of various one-way deterministic sensitivity analyses. Under all deterministic analyses explored, bevacizumab plus PRP remained the most cost-effective strategy based on the first scenario, while PRP alone remained the most cost-effective strategy based on the second scenario.

**Table 4:** Summary of probabilistic cost-effectiveness results of deterministic analyses using list prices.

Scenario	Treatment ranking best NMB^a^ [95% CI]	Treatment ranking 2nd best NMB^a^ [95% CI]	Treatment ranking 3rd best NMB^a^ [95% CI]
Initial treatment effects continued over a lifetime			
Treatment occurs in a separate visit to monitoring	Bevacizumab plus PRP £219,093 [£202,818– £236,071]	Bevacizumab £214,408 [£181,942– £238,641]	PRP £211,118 [£197,342– £225,083]
25% of patients continue to receive treatment after 5 years	Bevacizumab plus PRP £224,514 [£208,125– £240,548]	Bevacizumab £218,404 [£185,334– £241,027]	PRP £213,377 [£198,440– £227,677]
75% of patients continue to receive treatment after 5 years	Bevacizumab plus PRP £217,847 [£199,641– £234,758]	Bevacizumab £214,647 [£180,414– £238,490]	PRP £210,364 [£194,543– £224,992]
Natural history of PDR starts from 20 years	Bevacizumab plus PRP £220,706 [£205,919– £236,027]	Bevacizumab £216,369 [£185,853– £240,417]	PRP £212,110 [£198,536– £226,392]
Natural history of PDR starts from 10 years	Bevacizumab plus PRP £216,778 [£201,125– £230,910]	Bevacizumab £214,660 [£190,535– £233,035]	PRP £211,747 [£196,766– £224,943]
Natural history of PDR starts from 5 years	Bevacizumab plus PRP £212,037 [£197,137– £226,350]	PRP £211,822 [£196,994– £225,765]	Bevacizumab £211,592 [£191,279– £228,238]
Initial treatment effects stabilised over time (based on Protocol S)			
Treatment occurs in a separate visit to monitoring	PRP £223,103 [£209,587– £236,071]	Bevacizumab £218,061 [£203,668– £232,517]	Bevacizumab plus PRP £215,777 [£201,776– £228,853]
25% of patients continue to receive treatment after 5 years	PRP £225,945 [£212,843– £239,076]	Bevacizumab £222,310 [£208,716– £236,380]	Bevacizumab plus PRP £221,398 [£207,449– £234,926]
75% of patients continue to receive treatment after 5 years	PRP £221,831 [£208,929– £234,859]	Bevacizumab £217,149 [£201,980– £231,152]	Bevacizumab plus PRP £213,835 [£199,585– £227,237]
Natural history of PDR starts from 20 years	PRP £223,628 [£210,685– £236,826]	Bevacizumab £219,426 [£205,120– £234,198]	Bevacizumab plus PRP £217,409 [£203,854– £230,752]
Natural history of PDR starts from 10 years	PRP £223,965 [£210,297– £235,821]	Bevacizumab £219,817 [£205,539– £232,641]	Bevacizumab plus PRP £217,702 [£203,684– £230,781]
Natural history of PDR starts from 5 years	PRP £223,314 [£210,331– £237,609]	Bevacizumab £219,074 [£204,046– £233,466]	Bevacizumab plus PRP £217,046 [£203,786– £231,513]

*NMB* net monetary benefit, *PRP* panretinal photocoagulation, *QALYs* quality-adjusted life years.

^a^NMB was calculated as [(QALYs × £20,000) − Cost], the strategy with the maximum NMB is deemed to be the most cost-effective.

## Discussion

This Markov model-based study was conducted to inform the NICE guideline on diabetic retinopathy by assessing the lifetime cost-effectiveness of various anti-VEGFs and PRP (alone or in combination with anti-VEGF) for treating people with PDR without DMO based on two different treatment effect scenarios. The first scenario resulted in bevacizumab plus PRP being the most cost-effective strategy, whereby 1-year BCVA-based treatment effects from the NMA were applied for the first year and assumed to continue to be applied for the remainder of the lifetime for all interventions. This first scenario, however, might have overestimated treatment effects since some people may discontinue treatments due to a lack of effectiveness. On the other hand, assuming treatment effects stop immediately after treatment discontinuation might be incorrect as vision loss may happen gradually over time.

The second scenario resulted in PRP being the most cost-effective strategy in which 1-year BCVA-based treatment effects from the NMA were applied for the first year for all interventions, and then stabilised over time based on the 5-year outcomes reported in Protocol S [6]. For PRP, visual acuity stabilised beyond the first year since PRP can provide long-term stability for up to 15 years [5]. For anti-VEGFs, visual acuity stabilised beyond the second year after assuming a slow linear decline between the first and second years [6]. Given the assumption of stability in visual acuity, it may be incorrect to assume that people who switch their treatment beyond the first year for PRP and second year for anti-VEGFs would not receive any treatment benefit from switching. Protocol S only compared ranibizumab with PRP [6, 7]; hence, it is uncertain whether all anti-VEGFs would follow exactly the same disease progression pattern as ranibizumab.

Results were associated with large uncertainty due to wide confidence intervals around the BCVA-based treatment effects of anti-VEGFs compared with PRP from the NMA, particularly those of bevacizumab with or without PRP which was based on RCTs with small sample size and high risk of bias overall [25]. Four RCTs (three in Pakistan and one in Iran) compared bevacizumab plus PRP with PRP alone, while only one RCT in Jordan/Syria compared bevacizumab alone with PRP alone. Furthermore, bevacizumab does not have a market authorisation for use in ophthalmology conditions in the UK and must be reconstituted from the 100 mg vial into individual 1.25 mg doses in a specialist aseptic pharmacy environment.

The current model did not include a risk of developing DMO since most available studies report data on PDR and DMO separately, but any impact of developing DMO was expected to be captured within the BCVA-based transition probabilities. Even when DMO is reported as an adverse event, no differentiation was made between DMO requiring treatment and that which does not. This is consistent with the approach taken in previously published cost-effectiveness models [13, 19, 20, 22], although Walton et al. [10] allowed people with PDR to develop DMO within their model.

To achieve a better representation of real-life practice, the model allowed people to switch treatment between anti-VEGFs and PRP and no within therapeutic class switches were allowed. However, data on which to base the frequency and distributions of these subsequent treatments was limited. In addition, no data was available for the distribution of treatments for those switching from combination regimens. Furthermore, the model included some AEs and, although there is variation in reporting of AEs, this was unlikely to have impacted the conclusions drawn because the overall costs and disutilities associated with AEs included in the model were applied to a very small proportion of people. Mitchell et al. [20] did not include AEs as it was assumed to have a negligible impact on the cost-effectiveness. Patient time and productivity losses were not included in the analysis to align with the NICE reference case of NHS and PSS perspective in UK. Although these costs can represent a large burden to patients, how individual patient preferences are to be aggregated to a societal preference remains a theoretical and practical challenge. However, direct healthcare-related costs associated with low vision were applied to patients with BCVA ≤ 35 ETDRS letters by taking the costs of residential care, community care and low vision rehabilitation into account.

A two-eye model is likely to capture the cost implications of treatment more accurately given that PDR commonly affects both eyes, but the necessary data could not be obtained because most clinical trials report data for the study eye only. Aligned with previous literature [19, 20, 22], the model structure was based on one eye only but the costs associated with treatment of the second eye were included [23, 24]. Walton et al. [10] developed a two-eye model using DES, but it was not possible to use this type of structure given the absence of publicly available patient level data. Walton et al. [10] considered bilateral PDR at treatment initiation, which might have overestimated the costs and underestimated the QALYs of their analysis. Furthermore, Walton et al. [10] used utility values elicited from a DMO population using EQ-5D, which have been reported to be insensitive to QoL associated with changes in vision [19, 37, 38]. This study differed from Walton et al. [10] by including ranibizumab, aflibercept and bevacizumab separately rather than as a therapeutic class, making it possible to know the cost-effectiveness of a specific anti-VEGF.

The model presented here was developed in close collaboration with the clinical experts, and meetings were held at various stages of the model development phase to discuss the model structure and input parameters for clinical relevance, and to ensure that the model outputs were consistent and of clinical importance. The model coding was verified to detect any errors. Analysing different scenarios ensured varying parameter inputs had a feasible and hypothesised impact on the model outputs. The internal working of each model component during the model development phase was tested, e.g. an examination of the Markov trace was carried out by setting parameters in such a way that how the trace will look was anticipated.

Despite the limitations of a more simplified Markov model compared with DES, the main conclusion of this current study that anti-VEGF alone for treating PDR is unlikely to be cost-effective compared with PRP alone is in agreement with Walton et al. [10]. There were differences in the results such as the greater incremental QALY gain seen within this study, which may be explained by the review by Claxton et al. [39] that found the differences in outcomes between Markov and DES models become more apparent when bilateral disease is considered compared to unilateral.

There is a need for more empirical research to analyse the costs and benefits of alternative model structures to inform the same decision [40]; this study used a simple and flexible Markov model structure to make it more transparent and accessible for a range of stakeholders. A DES model is likely to be more suitable when the implementation of a defined model structure is not manageable as a Markov cohort model [40, 41], but this was not the case in structuring the BCVA-based health states. DES is a valuable modelling method for implementing more complex scenarios at the individual level for evaluating healthcare interventions, but this added realism should not result in more trust being placed in the model than is warranted [40]. The complexities of DES models are not borne solely by the model developers, but also by model users, particularly stakeholders who undertake detailed model reviews as part of the NICE decision-making process [40, 42]. Decision-makers need to be able to assess the quality of health economic models in line with scientific criteria of good practice [16], but this is difficult with complex models such as DES given the need of specialist analytical knowledge [40, 43, 44].

## Conclusions

PRP alone is likely to be more cost-effective compared with anti-VEGFs for treating people with PDR without DMO. However, the results should be interpreted with caution given the scarcity of long-term treatment outcomes in visual acuity and concern over bias in RCTs feeding the NMA. Although long-term follow-up in this population is challenging, the need for further research on long-term treatment outcomes in visual acuity and treatment patterns is evident to resolve uncertainties. This would then inform future cost-effectiveness models which should be of use to the decision-makers in terms of reviewing and implementing the models’ results timely and without any difficulties.

## Summary

### What was known before

- PDR is a leading cause of vision loss in the UK and the current standard of care is PRP. Although anti-VEGFs have been used in clinical trials, NICE has not yet recommended their use to treat PDR

### What this study adds

- Anti-VEGFs are unlikely to be cost-effective compared with PRP alone for treating people with PDR without DMO from a UK NHS perspective.
- Cost-effectiveness conclusions drawn from a Markov model used in this study are similar to those from a recently published discrete event simulation model.

## Supplementary information

Supplementary material
