# Supplementary material for: A Markov model assessing the cost-effectiveness of various anti-vascular endothelial growth factor drugs and panretinal photocoagulation for the treatment of proliferative diabetic retinopathy
Source: Eye (Lond). 2025 Feb 5;39(7):1364–72. doi: 10.1038/s41433-025-03641-4 (PMC12043813; doi:10.1038/s41433-025-03641-4)
Supplement: Supplementary file 1 — Supplementary material [file 41433_2025_3641_MOESM1_ESM.docx]

**SUPPLEMENTARY MATERIAL**

Table S1. Mean change over one year in best corrected visual acuity (BCVA) for panretinal photocoagulation (PRP)

| Study | No. of patients in PRP arm | Mean (SD) BCVA change in ETDRS | Mean (SD) BCVA change in LogMAR^a^ |
| --- | --- | --- | --- |
| CLARITY [1] | 102 | -2.90 (7.07^b^) | - |
| PRIDE [2] | 35 | -3.70 (17.10) | - |
| Weighted | - | -3.104 (10.585) | 0.062 (0.212) |

^a^ Change in LogMAR (logarithm of the minimum angle of resolution) is equivalent to -0.2 times the change in ETDRS (early treatment diabetic retinopathy study) letters, with an increase in letters and decrease in LogMAR both indicating improvement in vision.

^b^ SD (standard deviation) was calculated using reported SE (standard error) of 0.7

Table S2. Treatment effectiveness compared with panretinal photocoagulation (PRP)

| Treatment | Mean difference at 1 year in LogMAR  [95% CI] | Mean annual change in LogMAR | 3-monthly prob. of gaining one health state^a^ | 3-monthly prob. of losing one health state^a^ |
| --- | --- | --- | --- | --- |
| PRP | - | 0.062 | 4.79% | 8.48% |
| Aflibercept | -0.088  [-0.232 to 0.042] | -0.026 | 7.42% | 5.85% |
| Ranibizumab | -0.123  [-0.237 to -0.011] | -0.061 | 8.45% | 4.83% |
| Ranibizumab plus PRP | -0.080  [-0.163 to 0.003] | -0.018 | 7.18% | 6.09% |
| Bevacizumab | -0.193  [-1.172 to 0.786] | -0.131 | 10.07% | 3.06% |
| Bevacizumab plus PRP | -0.172  [-0.282 to -0.065] | -0.110 | 9.66% | 3.54% |

*^a^ Assumed that the change in BCVA (best corrected visual acuity) is normally distributed, mean difference was added to mean annual change before converting to 3-monthly transition probabilities of moving up or down by one health state; LogMAR (logarithm of the minimum angle of resolution).*

**Table S3.** Adverse events associated with treatment for proliferative diabetic retinopathy (PDR)

| Adverse events | PRP | Aflibercept | Ranibizumab | Ranibizumab plus PRP^a^ | Bevacizumab^a^ | Bevacizumab plus PRP^a^ |
| --- | --- | --- | --- | --- | --- | --- |
| Retinal detachment | 2.69% | 0.00% | 1.61% | 1.61% | 1.61% | 1.61% |
| Retinal tear | 0.00% | 0.22% | 0.00% | 0.00% | 0.00% | 0.00% |
| Vitreous haemorrhage | 4.31% | 0.00% | 7.64% | 7.64% | 7.64% | 7.64% |
| Increased intraocular pressure | 0.22% | 0.00% | 0.00% | 0.00% | 0.00% | 0.00% |
| Glaucoma | 0.75% | 0.00% | 0.40% | 0.40% | 0.40% | 0.40% |
| Endophthalmitis | 0.00% | 0.00% | 0.00% | 0.00% | 0.00% | 0.00% |
| Cataracts | 0.22% | 0.00% | 0.00% | 0.00% | 0.00% | 0.00% |
| Ocular pain | 0.87% | 1.32% | 0.00% | 0.00% | 0.00% | 0.00% |
| Stroke | 0.00% | 0.00% | 0.00% | 0.00% | 0.00% | 0.00% |
| Cardiovascular death | 0.00% | 0.00% | 0.00% | 0.00% | 0.00% | 0.00% |
| **Source** | Sivaprasad et al. [1]; Gross et al. [3] | Gross et al. [3] | Sivaprasad et al. [1] | Assumed same as ranibizumab | Assumed same as ranibizumab | Assumed same as ranibizumab |

*^a^ Due to a lack of data reported specifically for anti-VEGF use in PDR, adverse events for ranibizumab plus PRP, bevacizumab and bevacizumab plus PRP were assumed to be equivalent to ranibizumab; PRP (panretinal photocoagulation).*

Table S4. Cost of adverse events

| Resource | Cost | Probabilistic analysis^a^ | | Sources/Notes |
| --- | --- | --- | --- | --- |
|  |  | Distribution | Parameters |  |
| Retinal detachment | £2,314.220 | Gamma | μ=96.036  σ=24.097 | NG82 [4] assumed that 75% of patients require urgent vitrectomy (weighted average of non-elective long and short stay procedures, BZ84A-B major vitreous retinal procedures, 19 years and over, with CC score 0-2+) and 25% of patients have elective surgery (weighted average of day case procedures, BZ84A-B major vitreous retinal procedures, 19 years and over, with CC score 0-2+). |
| Retinal tear | £185.608 | Gamma | μ=96.036  σ=1.933 | BZ84A-B major vitreous retinal procedures. Total HRGs, weighted average of CC scores. |
| Vitreous haemorrhage | £482.840 | Gamma | μ=96.036  σ=5.028 | BZ86B intermediate vitreous retinal procedures, 19 years and over, with CC score 0-1, weighted average of non-elective long and short stay based on TA824 [5]. |
| Increased intraocular pressure | £1,012.241 | Gamma | μ=96.036  σ=10.540 | BZ24D-G non-surgical ophthalmology. Total HRGs, weighted average of CC scores, with and without interventions. |
| Glaucoma | £883.000 | Gamma | μ=96.036  σ=9.194 | Trabeculectomy BZ17B, service code 130 (ophthalmology), major glaucoma procedures, with CC score 0 (day case) based on evidence review group discussion for TA349 [6] that trabeculectomy is the main procedure used. |
| Endophthalmitis | £1,520.972 | Gamma | μ=96.036  σ=15.837 | Calculated using the distributions from the AMD (NG82 [4]) and cataract (NG77 [7]) guidelines. Assumed 18.31% patients require vitrectomy, 38.46% require urgent vitrectomies, and 17.95% patients require at least 1 revision, 5.13% of patients require 2 revisions as reported by Kamalarajah et al. [8]. All patients were assumed to require vitreous tap (weighted average of procedures BZ87A) based on NG82 [4] committee guidance. Elective vitrectomy assumed to be the weighted average of elective and day case procedures for BZ84A-B major vitreous retinal procedures, urgent vitrectomy assumed to be the weighted average of nonelective long-stay procedures BZ84A-B. It was assumed 5.5 outpatient visits will be needed based on NG82 [4] consultant led non-admitted face-to-face attendance. Code 130 (ophthalmology). It was also assumed that all patients require medication of Amikacin 500mg/2ml. |
| Cataracts | £1,945.466 | Gamma | μ=96.036  σ=20.258 | AMD (NG82 [4]) and cataract (NG77 [7]) guidelines, weighted average of non-elective short stay and day case codes for phacoemulsification cataract extraction and lens implant with CC score 4+, 2-3, 0-1, BZ34A-C. |
| Ocular pain | £1,012.241 | Gamma | μ=96.036  σ=10.540 | BZ24D-G non-surgical ophthalmology. Total HRGs, weighted average of CC scores, with and without interventions. |
| Stroke | £3,655.557 | Gamma | μ=96.036  σ=38.064 | AA35A-F, stroke. Total HRGs, weighted average of CC scores. |
| Cardiovascular death | £598.624 | Gamma | μ=96.036  σ=6.233 | VB99Z, emergency medicine, patient dead on arrival. |
| Myocardial infarction | £1,596.387 | Gamma | μ=96.036  σ=16.623 | EB10A-E actual or suspected myocardial infarction. Total HRGs, weighted average of CC scores. |

*^a^ Varied by ±20% where relevant data were not available. AMD (age-related macular degeneration); HRGs (Healthcare Resource Groups).*

Table S5. Number of monitoring visits

| Treatment | Year 1 | Year 2 | Year 3 | Year 4 | Year 5 onwards |
| --- | --- | --- | --- | --- | --- |
| PRP | 3.063 | 2.625 | 1.000 | 1.000 | 1.000 |
| Aflibercept | 12.000 | 7.755 | 4.518 | 2.909 | 2.182 |
| Ranibizumab | 12.000 | 7.755 | 4.518 | 2.909 | 2.182 |
| Ranibizumab plus PRP | 12.000 | 7.755 | 4.518 | 2.909 | 2.182 |
| Bevacizumab | 12.000 | 7.755 | 4.518 | 2.909 | 2.182 |
| Bevacizumab plus PRP | 12.000 | 7.755 | 4.518 | 2.909 | 2.182 |
| **Source** | **PRP:** average of Royle et al. [9], Maredza et al. [10], Lois et al. [11] and Gross et al. [3]  **Anti-VEGFs:** Gross et al. [3] | **PRP:** average of Lois et al. [11] and Gross et al. [3]  **Anti-VEGFs:** average of DMO literature^a^ | **PRP:** Lois et al. [11]  **Anti-VEGFs:** average of DMO literature^a^ | **PRP:** Lois et al. [11]  **Anti-VEGFs:** average of DMO literature^a^ | **PRP:** Lois et al. [11]  **Anti-VEGFs:** average of DMO literature^a^ |

^a^ Due to a lack of data for the proliferative diabetic retinopathy population, the model assumed average of anti-VEGF visits across ranibizumab and aflibercept based on diabetic macular oedema literature for year 2 onwards; PRP (panretinal photocoagulation).

Table S6. Number of injections

| Treatment | Year 1 | Year 2 | Year 3 | Year 4 | Year 5 onwards |
| --- | --- | --- | --- | --- | --- |
| PRP | 0 | 0 | 0 | 0 | 0 |
| Aflibercept | 6.900^a^ | 3.300^a^ | 1.650 | 1.238 | 1.176 |
| Ranibizumab | 6.900 | 3.300 | 1.650 | 1.238 | 1.176 |
| Ranibizumab plus PRP | 6.900^a^ | 3.300^a^ | 1.650 | 1.238 | 1.176 |
| Bevacizumab | 6.900^a^ | 3.300^a^ | 1.650 | 1.238 | 1.176 |
| Bevacizumab plus PRP | 6.900^a^ | 3.300^a^ | 1.650 | 1.238 | 1.176 |
| **Source** | Gross et al. [3] | Gross et al. [3] | Assumed 50% decrease from previous year^b^ | Assumed 25% decrease from previous year^b^ | Assumed 5% decrease from previous year^b^ |

^a^ Assumed same number of injections (years 1 and 2) for all anti-VEGFs as ranibizumab based on clinical consensus.

^b^ Assumed a percentage decrease in injection frequency from the previous year for year 3 onwards based on the relationship identified in decrease over time from anti-VEGF use in diabetic macular oedema.

Table S7. Number of panretinal photocoagulation (PRP) treatments

| Treatment | Year 1 | Year 2 | Year 3 | Year 4 | Year 5 onwards |
| --- | --- | --- | --- | --- | --- |
| PRP | 1.815 | 0.689 | 0.689 | 0.689 | 0.689 |
| Aflibercept | - | - | - | - | - |
| Ranibizumab | - | - | - | - | - |
| Ranibizumab plus PRP^a^ | 1.815 | 0.689 | 0.689 | 0.689 | 0.689 |
| Bevacizumab | - | - | - | - | ]- |
| Bevacizumab plus PRP^a^ | 1.815 | 0.689 | 0.689 | 0.689 | 0.689 |
| **Source** | Average of Sivaprasad et al. [1] and Gross et al. [3] | Gross et al. [3],  45% required additional PRP | Assumed same as year 2 (clinical consensus) | Assumed same as year 2 (clinical consensus) | Assumed same as year 2 (clinical consensus) |

^a^ Assumed the same number of PRP treatments across all treatments in combination with PRP.

Table S8. Utility losses associated with adverse events

| Resource | Utility decrement | Event duration | Source |
| --- | --- | --- | --- |
| Retinal detachment | 0.270 | 3 months | NG82 [4] |
| Retinal tear | 0.000 | Immediate repair | NG82 [4] |
| Vitreous haemorrhage | 0.020 | - | TA346 [12]; Pochopien et al. [13] |
| Increased intraocular pressure | 0.000 | - | TA346 [12]; Pochopien et al. [13] |
| Glaucoma | 0.000 | - | TA346 [12]; Pochopien et al. [13] |
| Endophthalmitis | 0.300 | 20% experience 1-year quality-of-life effect and 80% 1.5 months | NG82 [4] |
| Cataracts | 0.142 | 1 month | NG82 [4] |
| Stroke | 0.000 | - | No information identified |
| Cardiovascular death | 0.000 | - | No information identified |
| Myocardial infarction | 0.000 | - | No information identified |
| Injection related anxiety | 0.071 | 1 day | TA613 [14]; Dolan et al. [15] |

Table S9. Distribution of subsequent treatment

| To\From^a^ | PRP^b^ | Aflibercept | Ranibizumab | Bevacizumab |
| --- | --- | --- | --- | --- |
| No treatment | 95.0% | 94.0% | 94.0% | 94.0% |
| PRP | 0.0% | 6.0% | 6.0% | 6.0% |
| Aflibercept | 3.5% | 0.0% | 0.0% | 0.0% |
| Ranibizumab | 1.0% | 0.0% | 0.0% | 0.0% |
| Ranibizumab plus PRP^a^ | 0.0% | 0.0% | 0.0% | 0.0% |
| Bevacizumab | 0.5% | 0.0% | 0.0% | 0.0% |
| Bevacizumab plus PRP^a^ | 0.0% | 0.0% | 0.0% | 0.0% |
| **Source** | Clinical consensus | Assumed same as ranibizumab | Gross et al. [3] | Assumed same as ranibizumab |

*^a^ People initially on combination treatment were assumed to receive no further treatment.*

*^b^ Distribution of subsequent treatment for panretinal photocoagulation (PRP) was based on clinical opinion assuming only 5% of people on PRP would require anti-VEGF treatment, of which it was assumed 70% of those having anti-VEGF would have aflibercept, 20% ranibizumab and 10% bevacizumab.*

Table S10. Cost of subsequent treatment by first line regimen

| First line regimen | Cost of subsequent treatment |
| --- | --- |
| Panretinal photocoagulation (PRP) | £483.28 |
| Aflibercept | £25.34 |
| Ranibizumab | £25.34 |
| Ranibizumab plus PRP | - |
| Bevacizumab | £25.34 |
| Bevacizumab plus PRP | - |

**References**

1. Sivaprasad S, Prevost AT, Vasconcelos JC, Riddell A, Murphy C, Kelly J, et al. Clinical efficacy of intravitreal aflibercept versus panretinal photocoagulation for best corrected visual acuity in patients with proliferative diabetic retinopathy at 52 weeks (CLARITY): a multicentre, single-blinded, randomised, controlled, phase 2b, non-inferiority trial. The Lancet. 2017;389(10085):2193-203.

2. Lang GE, Stahl A, Voegeler J, Quiering C, Lorenz K, Spital G, et al. Efficacy and safety of ranibizumab with or without panretinal laser photocoagulation versus laser photocoagulation alone in proliferative diabetic retinopathy–the PRIDE study. Acta ophthalmologica. 2020;98(5):e530-e9.

3. Gross JG, Glassman AR, Jampol LM, Inusah S, Aiello LP, Antoszyk AN, et al. Panretinal photocoagulation vs intravitreous ranibizumab for proliferative diabetic retinopathy: a randomized clinical trial. Jama. 2015;314(20):2137-46.

4. National Institute for Health and Care Excellence (NICE). Age-related macular degeneration. NICE guideline NG82. 2018. Available from: <https://www.nice.org.uk/guidance/ng82>.

5. National Institute for Health and Care Excellence (NICE). Dexamethasone intravitreal implant for treating diabetic macular oedema. Technology appraisal guidance TA824. 2022. Available from: <https://www.nice.org.uk/guidance/ta824>.

6. National Institute for Health and Care Excellence (NICE). Dexamethasone intravitreal implant for treating diabetic macular oedema. Technology appraisal guidance A349. 2015. Available from: <https://www.nice.org.uk/guidance/ta349>.

7. National Institute for Health and Clinical Excellence (NICE). Cataracts in adults: management. NICE guideline NG77. 2017. Available from: <https://www.nice.org.uk/guidance/ng77>.

8. Kamalarajah S, Silvestri G, Sharma N, Khan A, Foot B, Ling R, et al. Surveillance of endophthalmitis following cataract surgery in the UK. Eye. 2004;18(6):580-7.

9. Royle P, Mistry H, Auguste P, Shyangdan D, Freeman K, Lois N, et al. Pan-retinal photocoagulation and other forms of laser treatment and drug therapies for non-proliferative diabetic retinopathy: systematic review and economic evaluation. Health Technology Assessment. 2015;19(51):1-248.

10. Maredza M, Mistry H, Lois N, Aldington S, Waugh N. Surveillance of people with previously successfully treated diabetic macular oedema and proliferative diabetic retinopathy by trained ophthalmic graders: cost analysis from the EMERALD study. British Journal of Ophthalmology. 2022;106(11):1549-54.

11. Lois N, Cook JA, Wang A, Aldington S, Mistry H, Maredza M, et al. Evaluation of a new model of care for people with complications of diabetic retinopathy: the EMERALD study. Ophthalmology. 2021;128(4):561-73.

12. National Institute for Health and Care Excellence (NICE). Aflibercept for treating diabetic macular oedema. Technology appraisal guidance TA346. 2015. Available from: <https://www.nice.org.uk/guidance/ta346>.

13. Pochopien M, Beiderbeck A, McEwan P, Zur R, Toumi M, Aballéa S. Cost-effectiveness of fluocinolone acetonide implant (ILUVIEN®) in UK patients with chronic diabetic macular oedema considered insufficiently responsive to available therapies. BMC Health Services Research. 2019;19:1-14.

14. National Institute for Health and Clinical Excellence (NICE). Fluocinolone acetonide intravitreal implant for treating chronic diabetic macular oedema in phakic eyes after an inadequate response to previous therapy. Technology appraisal guidance TA613. 2019. Available from: <https://www.nice.org.uk/guidance/ta613>.

15. Dolan P. Modeling valuations for EuroQol health states. Medical care. 1997;35(11):1095-108.
